# Topographic associations of hyperreflective materials in diabetic retinopathy: a multimodal correlation with microvascular pathology, structural remodeling and systemic metabolic dysregulation

**DOI:** 10.3389/fmed.2025.1619819

**Published:** 2025-07-16

**Authors:** Lan Zhou, Hongyan Sun, Cunzi Li, Tianyi Luo, Ting Meng, Xiaojun Wen, Zilin Chen, Yan Hu, Ming-Ming Yang

**Affiliations:** ^1^Department of Ophthalmology, Shenzhen People's Hospital (The First Affiliated Hospital, Southern University of Science and Technology, The Second Clinical Medical College, Jinan University), Shenzhen, China; ^2^Post-doctoral Scientific Research Station of Basic Medicine, Jinan University, Guangzhou, China; ^3^The Second Clinical Medical College of Jinan University (Shenzhen People's Hospital), Guangzhou, China; ^4^Ophthalmological Center of Huizhou Central People's Hospital, Huizhou, China; ^5^School of Computer Science and Engineering, Southern University of Science and Technology, Shenzhen, China

**Keywords:** hyperreflective materials, optical coherence tomography angiography, diabetic retinopathy, diabetic macular edema, lipid metabolism

## Abstract

**Background:**

Hyperreflective materials (HRMs), enigmatic biomarkers observed in diabetic retinopathy (DR), exhibit poorly characterized pathophysiological origins and clinical implications.

**Methods:**

This retrospective cross-sectional study investigates the spatial distribution patterns of HRMs subtypes and their integrative relationships with retinal microvascular architecture, structural remodeling, and systemic metabolic parameters in 205 DR eyes. HRMs were systematically classified via multimodal optical coherence tomography angiography (OCTA) analysis, incorporating topographic localization (inner vs. outer retinal), reflectivity profiles, morphometric dimensions, posterior shadowing artifacts, and decorrelation signal. Quantitative correlations were established between HRMs subtypes and OCTA-derived vascular parameters (intraretinal microvascular abnormalities [IRMA], non-perfusion [NP] areas, microaneurysms), diabetic macular edema (DME) status, and systemic metabolic indices (glycemic control, lipid profiles, renal function, inflammatory markers).

**Results:**

Six distinct HRMs phenotypes were identified: inner retinal hyperreflective spots (IRHFs), outer retinal hyperreflective spots (ORHFs), intraretinal hard exudates (IRHE), outer retinal hard exudates (ORHE), decorrelation-positive HRMs, and cotton-wool spots. Spatial mapping revealed predominant HRMs colocalization with IRMA territories (75.4% IRHFs, 89.5% ORHFs, 90.8% IRHE, 94% ORHE), while 19% of IRHFs and 8.7% of ORHFs overlapped NP zones. Decorrelation-positive HRMs demonstrated dual associations with IRMA (77.6%) and microaneurysms (21.0%). DME eyes exhibited significantly elevated HRMs density within IRMA and NP regions (*P* < 0.001). Multivariate analysis identified dyslipidemia as a strong predictor of HRMs burden.

**Conclusions:**

These findings establish HRMs as spatially resolved biomarkers of diabetic retinal pathophysiology, reflecting compartment specific interactions between microvascular incompetence (IRMA-associated barrier failure), ischemic remodeling (NP zones), and systemic metabolic dysregulation. The colocalization of HRMs subtypes with IRMA walls and leakage-prone microaneurysms supports their putative role as optical signatures of lipoprotein extravasation and inflammatory lipidotoxicity in DR progression.

## Background

Hyperreflective materials (HRMs), alternatively termed hyperreflective foci or spots, represent distinct optical coherence tomography (OCT) findings characterized by intraretinal or subretinal punctate hyperreflectivity (1). These entities are observed in both physiological retinal conditions and various retinopathies, yet their precise definition, anatomical localization, cellular origin, and clinical significance remain subjects of ongoing debate due to their heterogeneous morphological presentations across retinal layers and disease states. In physiological contexts, HRMs typically manifest as discrete inner retinal punctate changes with reflectivity comparable to the nerve fiber layer, though their etiological basis remains undetermined (2, 3). The pathophysiological interpretation becomes more complex in disease states: Age-related macular degeneration (AMD) studies present conflicting evidence, with histopathological correlations suggesting HRMs may represent either migrated retinal pigment epithelium (RPE) cells (4, 5), activated microglia (1, 6) or lipid-engorged monocytes (7, 8). Diabetic retinopathy (DR) investigations reveal even greater diagnostic ambiguity. Spectral-domain OCT (SD-OCT) classifications based on size, location, and posterior shadowing characteristics have led to proposed associations with microglial activation, hard exudates, and microaneurysms (9–11). Alternative hypotheses implicate photoreceptor degeneration byproducts (12) or lipoprotein extravasation (13) in HRMs formation. The pathobiological significance of HRMs in diabetic macular edema (DME) remains a subject of ongoing debate, particularly regarding their fundamental role as primary drivers of inflammatory cascades vs. secondary epiphenomena resulting from chronic edema. This controversy extends to their clinical utility as prognostic biomarkers in anti-VEGF therapy, where disparate findings emerge from contemporary studies–while some report significant correlations between HRMs resolution and visual acuity improvement (14), others demonstrate no such association (15). More fundamentally, current research paradigms exhibit two critical limitations in characterizing HRMs within diabetic retinopathy (DR): First, insufficient elucidation of their vascular-metabolic interactions, a crucial oversight given DR's pathognomonic manifestation as a microvascular disorder stemming from systemic glucose dysregulation. Second, persistent ambiguity persists regarding their cellular lineage and ontogenetic pathways. These dual knowledge deficits necessitate the implementation of multimodal imaging technologies to systematically investigate HRMs pathobiology through the integrated lens of metabolic dyshomeostasis and vascular pathophysiology.

The advent of optical coherence tomography angiography (OCTA) provides a critical technological bridge, enabling simultaneous non-invasive visualization of retinal vasculature and HRMs topography. Leveraging this dual imaging capability, our study systematically investigates: (1) spatial concordance between HRMs distribution and vascular abnormalities in DR. (2) HRMs-macular edema interactions across disease stages. (3) Metabolic correlates of HRMs burden, particularly in relation to glycemic control markers.

## Materials and methods

### Subjects

This is a retrospective, cross-sectional study performed on 105 patients diagnosed with DR by the Department of Ophthalmology of Shenzhen People's Hospital between January 2020 and May 2023. All the research and measurements were conducted in compliance with the tenets of the Declaration of Helsinki. The study was approved by the ethics committee of the hospital and informed consent was obtained from all individuals after a detailed discussion of the nature and possible consequences of the study procedures.

All participants underwent standardized multimodal ophthalmic evaluations including: slit-lamp biomicroscopy examination, Fundus Fluorescein Angiography (Topcon, TRC 50IA; Tokyo, Japan), OCTA (Zeiss, CIRRUS AngioPlex, Dublin, CA). Demographic and clinical factors including age, sex, history of hypertension, and serum lipid profile, glucose metabolism, leukocytes number, duration of diabetes and the grade of diabetic retinopathy were also reviewed. Inclusion criteria were as follows: (1) age above 18 years; (2) type 1 or 2 diabetes mellitus; and (3) the grade of DR was based on the international clinical diabetic retinopathy disease severity scale and DME was based on international clinical diabetic macular edema disease severity scale proposed by American Academy of Ophthalmology in 2019. Exclusion criteria were: (1) presence of any other retinal disorder such as retinal vein occlusion, retinal detachment, uveitis or any other maculopathy; (2) other ocular condition that compromises media opacities (i.e., vitreous hemorrhage or mature cataract); (3) previous treatment with any intraocular surgery, laser photocoagulation or intravitreal injection of anti-VEGF or corticosteroids drug; (4) clearly identify patients taking lipid-lowering drugs such as fenofibrate, statins et al.; and (5) poor quality OCTA images defined by the signal strength index of <5/10.

### Optical coherence tomography acquisition and processing

Macular-centered 6 × 6 mm scan grids (350 × 350 A-scan density) were acquired using Cirrus 5000 Angioplex. Optical microangiography algorithms generated three-dimensional vascular maps through semi-automated segmentation (AngioPlex Metrix v10.0, an integrated analytical software suite for Cirrus 5000 Angioplex and available through two primary channels: directly via the OCT imaging system or through the ZEISS FORUM platform), which is comprise of structural OCT B-scans with flow overlays, superficial/deep vascular plexus en face projections and depth-resolved capillary density maps. Vascular layers were segmented using validated anatomical landmarks: the superficial vascular plexus was defined as extending from the internal limiting membrane to 9 μm above the inner plexiform layer-inner nuclear layer (IPL-INL) junction, and the deep vascular complex encompassed the region from 9 μm above the IPL-INL junction to 9 μm below the outer plexiform layer-outer nuclear layer (OPL-ONL) junction. Corresponding structural en face OCT images were analyzed using identical segmentation slabs to ensure anatomical correspondence. The walls of cystoid spaces are typically formed by adjacent parenchyma or thin, highly reflective lines resulting from optical property differences at the boundaries following hyporeflective fluid accumulation. The border of a serous retinal detachment is defined as the demarcation zone between the detached neurosensory retina and RPE, with underlying hyporeflective subretinal fluid causing an abrupt optical reflectivity transition. All automated segmentations underwent rigorous quality assessment: (1) B-scans with flow overlay were systematically reviewed to identify HRMs or hyperreflective spots exhibiting decorrelation signals; (2) segmentation accuracy was verified through dynamic B-scan navigation using orthogonal (horizontal/vertical) reference lines; and (3) manual corrections were applied when segmentation errors were detected, particularly at layer boundary transitions.

### Vascular pathology quantification

Four distinct categories of pathological microvascular alterations detectable by OCT angiography (OCTA) were analyzed and their morphological classifications were established based on well-characterized criteria from prior validated studies (16, 17). Specifically: (1) microaneurysms were defined as focal capillary dilations (25–100 μm diameter) appearing as hyperreflective dots on OCTA, corresponding to the deep-red punctate lesions observed clinically via ophthalmoscopy; (2) intraretinal microvascular abnormalities (IRMAs) were identified as aberrant, tortuous microvascular structures exhibiting abnormal arteriolar-venular connections within the retinal layers, distinguished by their characteristic branching pattern without vitreal protrusion; (3) retinal neovascular membranes were characterized by the presence of abnormal blood flow signals above the inner limiting membrane, indicating pathological angiogenesis; and (4) non-perfusion area manifested as discrete regions of capillary dropout with complete absence of detectable vascular flow on OCTA imaging.

### Image grading of HRMs phenotyping

Two independent masked retinal specialists (L.Z. and H.S.) performed qualitative and quantitative assessments of HRMs using registered B-scan and en face OCT/OCTA images. A senior retinal specialist (M.M.Y.) served as adjudicator for discordant cases to ensure consensus. Each HRMs was evaluated based on the following standardized criteria: (1) anatomical location [inner retina defined as layers between inner limiting membrane and inner nuclear layer, outer retina defined as layers below the inner boundary of the outer nuclear layer (18)], (2) size (≥30 μm or ≤ 30 μm), (3) presence or absence of back shadowing, (4) reflectivity [two levels of hyperreflectivity were identified: moderate reflectivity similar to normal NFL and high reflectivity similar to RPE-Bruch membrane (18)], (5) visible or invisible on structural en face images, and (6) decorrelation signal (9). For systematic analysis, we employed a standardized localization method: (1) each HRMs was sequentially identified across 350 linear B-scans. (2) Precursor cursor placement on B-scan images enabled: exact HRMs localization in the *z*-axis and automated co-registration with corresponding en face OCTA maps. (3) This spatial mapping protocol ensured precise correlation between cross-sectional structural features (B-scan) and microvascular characteristics (en face OCTA).

### Statistics

Inter-rater reliability was quantified via intraclass correlation coefficients (ICCs) with 95% confidence intervals. To examine the association between HRMs subtypes and retinal vascular pathology, we performed the following analyses: (1) the Kruskal–Wallis test was employed to compare HRMs counts across the four predefined categories of abnormal vascular morphology; (2) *Post-hoc* pairwise comparisons with Dunn–Bonferroni correction were conducted for significant findings. Generalized estimating equations (GEE) with exchangeable correlation matrices were implemented to address clustered data structures in two analytical contexts: (1) comparative analysis of HRMs burden between DME (+) and DME (–) cohorts, (2) systematic evaluation of HRMs characteristics against seven metabolic covariates including diabetes duration, hypertension status, DR severity (graded per AAO criteria), glucose homeostasis markers [fasting blood glucose (FBG), HbA1c], lipid profile parameters (TG, TC, HDL, LDL, and APO-A/B), systemic inflammation (leukocyte count), and renal function (urinary albumin-creatinine ratio, UACR). (3) Correlation of HRMs with visual function. All analyses were performed using SPSS v27 (IBM) with statistical significance set at *P* < 0.05 (two-tailed). Effect sizes with 95% confidence intervals are reported for significant associations.

## Results

### Demographics and ocular characteristics of the whole cohort of patients

The study cohort comprised 105 treatment-naïve diabetic retinopathy (DR) patients (69 males, 36 females; mean age 55.0 ± 8.1 years) undergoing standardized OCTA imaging, yielding 205 evaluable eyes (104 right, 101 left). Table 1 summarizes the demographic characteristics. DR severity stratification revealed: 21.5% (44/205) mild non-proliferative DR (NPDR), 11.2% (23/205) moderate NPDR, 36.6% (75/205) severe NPDR, and 30.7% (63/205) proliferative DR (PDR). Diabetic macular edema (DME) was present in 52.2% (107/205) of eyes, with morphological subtypes distributed as: cystoid (21.0%, 23/107), mixed pattern (23.9%, 25/107), diffuse (6.3%, 7/107), and serous (1.0%, 1/107).

**Table 1:** Baseline demographics and ocular characteristics of the whole cohort of patients with hyperreflective materials detected on optical coherence tomography angiography.

**Study population**	**(*n* = 105)**
Age (years), mean ± SD	55 ± 8
Gender male, *n* (%)	69 (65.7)
DM duration (years), mean ± SD	8.8 ± 5.3
With hypertension, *n* (%)	59 (56.2)
FBG	11.0 ± 5.4
HbA1c (%), mean ± SD	9.2 ± 2.2
**Ocular characteristic**	
No. of eyes	205
Right eye	104
Left eye	101
**DR severity, no. of eyes (%)**	
Mild NPDR	44 (21.5)
Moderate NPDR	23 (11.2)
Severe NPDR	75 (36.6)
Proliferative DR	63 (30.7)
**DR with DME, no. of eyes (%)**	
Diffuse	13 (6.3)
Cystoid	43 (21.0)
Serous	2 (1.0)
Mixed	49 (23.9)
**Visual acuity, LogMAR, mean** ±**SD**	
DR with DME	0.7 ± 0.5
DR without DME	0.2 ± 0.2
**Central subfield thickness (**μ**m), mean** ±**SD**	
DR with DMEs	371.4 ± 128.4
DR without DME	220 ± 17.9

DR, diabetic retinopathy; NPDR, non-proliferative diabetic retinopathy; DME, diabetic macular edema; LogMAR, logarithm of the minimal angle of resolution.

### Classification of hyperreflective materials subtypes

**Figure 1:**
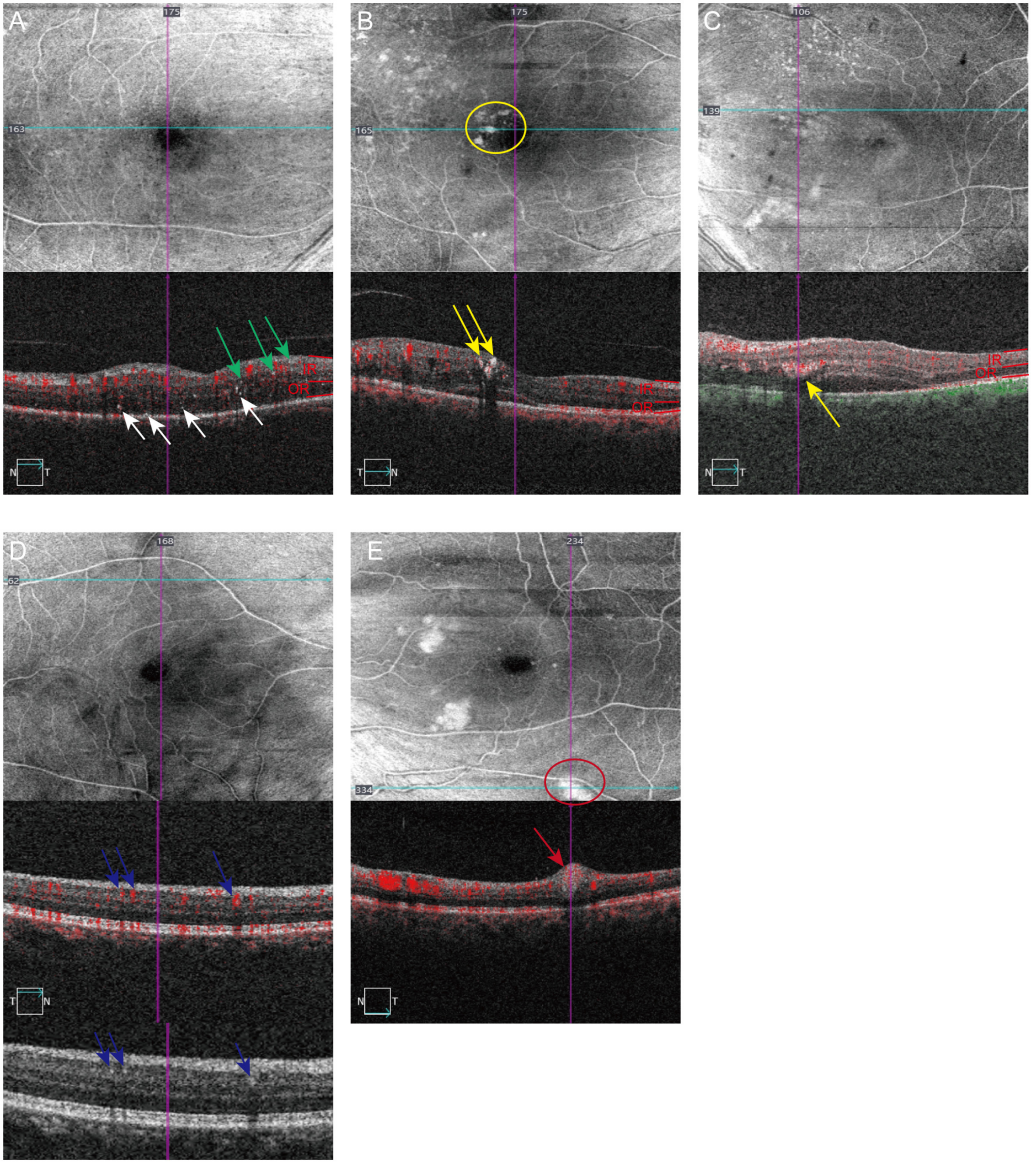
Category of hyperreflective materials based on characters including location, reflectivity, size and presence or absence of back shadowing, decorrelation signal on B-scan and structural en face images (6 × 6 mm scanning area) using OCTA. The scanning position of each image is identified by horizontal (lake blue) or vertical navigation (purple) line. IR is defined as layers between the inner limiting membrane and the OPL; OR is defined as the Henle NFL and outer nuclear layer. **(A)** Inner retinal hyperreflective spots (IRHFs) and outer retinal hyperreflective spots (ORHFs), invisible on structural en-face images, are observed on B-scan OCTA images characterized by size small than 30 μm, reflectivity similar to NFL, not forming any back shadowing and without decorrelation signal. Green arrows indicating IRHFs and white arrows indicating ORHFs. **(B)** Inner retinal hard exudates (IRHE, yellow arrows) are characterized by dimension >30 mm, reflectivity similar to RPE with back shadowing, obviously seen on en face images (yellow circle). **(C)** Outer retinal hard exudates (ORHE, yellow arrows) located in the outer retina, share the similar character as IRHE but invisible on superficial en face images. **(D)** Decorrelation-positive HRMs (blue arrows) are visible on B-scan OCTA images with apparent flow signal (in red), dimension >30 mm, reflectivity similar to NFL with back shadowing. Detailed structure can be seen on OCT B scan. **(E)** Cotton-wool spots, marked with red circles on en face and red arrows on B scan OCTA images, are located on NFL with large size, medium reflectivity and no back shadowing.

**Figure 2:**
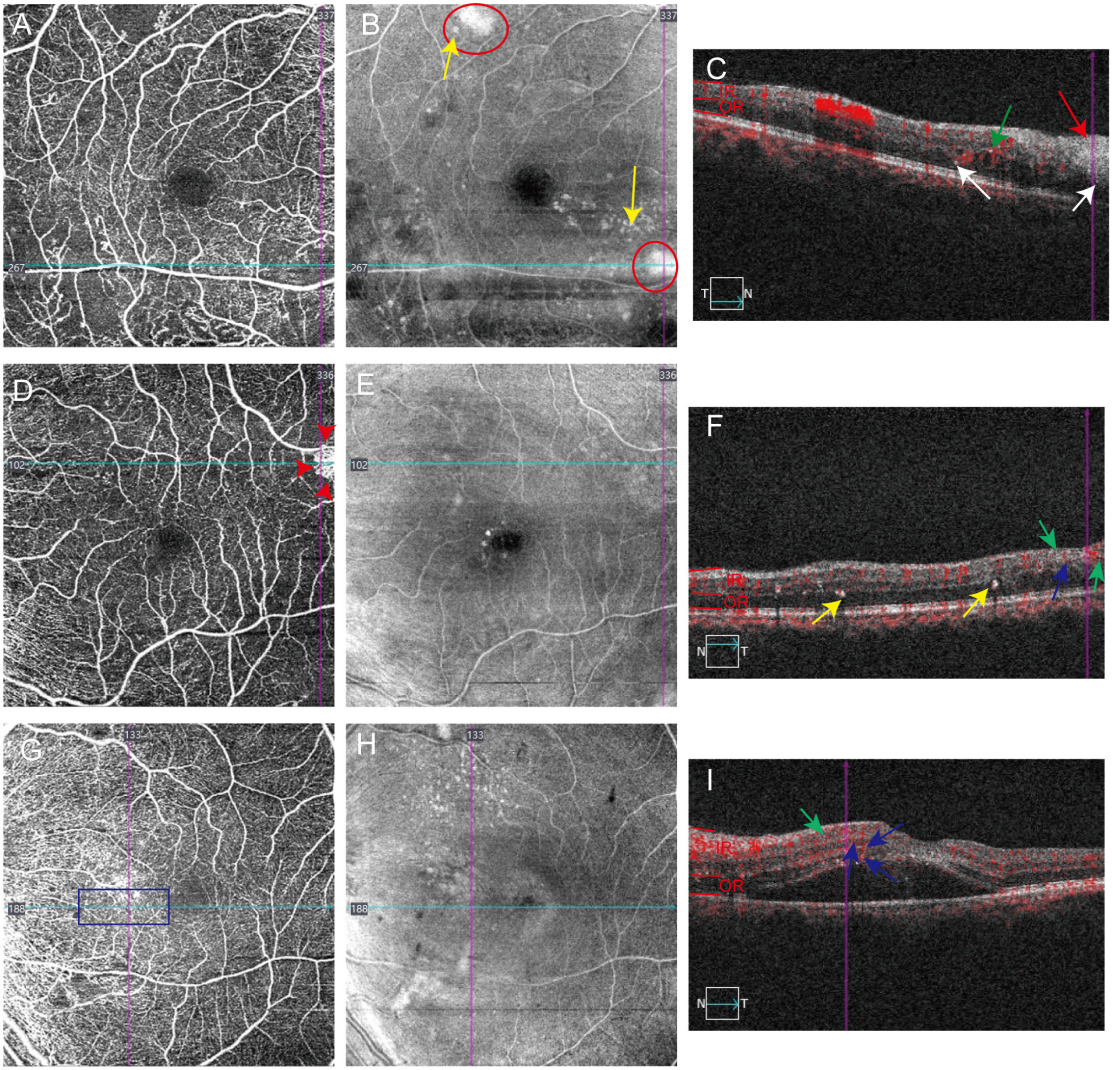
Association of hyperreflective materials distribution with vascular abnormalities including no perfusion area, neovascular membrane and microneurysm. **(A–C)** No perfusion is observed on superficial vascular plexuses en face OCTA images **(A)**, where cotton-wool spots (**B**, red circles; **C**, red arrows), hard exudates (**B**, yellow arrows), IRHFs (**C**, green arrows) and ORHFs (**C**, white arrows) are visible. **(D–F)** IRHFs (**F**, green arrows) and decorrelation-positive hyperreflective materials (**F**, blue arrows) are distributed in the neovascular area detected by superficial vascular plexuses en face OCTA images **(D)**. **(G–I)** IRHFs (**I**, green arrows) and decorrelation-positive hyperreflective materials (**I**, blue arrows) were located in the area of microneurysm (**G**, blue rectangle).

Six distinct HRMs subtypes were systematically classified through multimodal image analysis (Figure 1), defined by anatomical localization, morphometrics, and signal characteristics: (1) inner retinal hyperreflective spots (IRHFs, inner retina, size ≤ 30 μm, reflectivity similar to nerve fiber layer or RPE, invisible on en face images, absence of back shadowing, Figure 1A green arrow); (2) outer retinal hyperreflective spots (ORHFs, outer retina, size ≤ 30 μm, reflectivity similar to RPE or nerve fiber layer, invisible on en face images, absence of back shadowing, Figure 1A white arrows); (3) inner retinal hard exudates (IRHE, inner retina, size >30 μm, hyperreflective, visible on en face images, presence of back shadowing, Figure 1B yellow circle and arrows); (4) outer retinal hard exudates (ORHE, similar to IRHE but located in outer retina and invisible on superficial en face images, Figures 1C, 2E, F, yellow arrows); (5) decorrelation-positive HRMs (size>30 μm, hyperreflective, invisible on en face images, presence of back shadowing and non-vascular decorrelation signal, Figure 1D blue arrows); (6) cotton-wool spot (size>30 μm, located in the RNFL, hyperreflective, absence of back shadowing, Figure 1E red circle and arrows).

### Spatial distribution of hyperreflective materials relative to retinal vascular abnormalities

**Figure 3:**
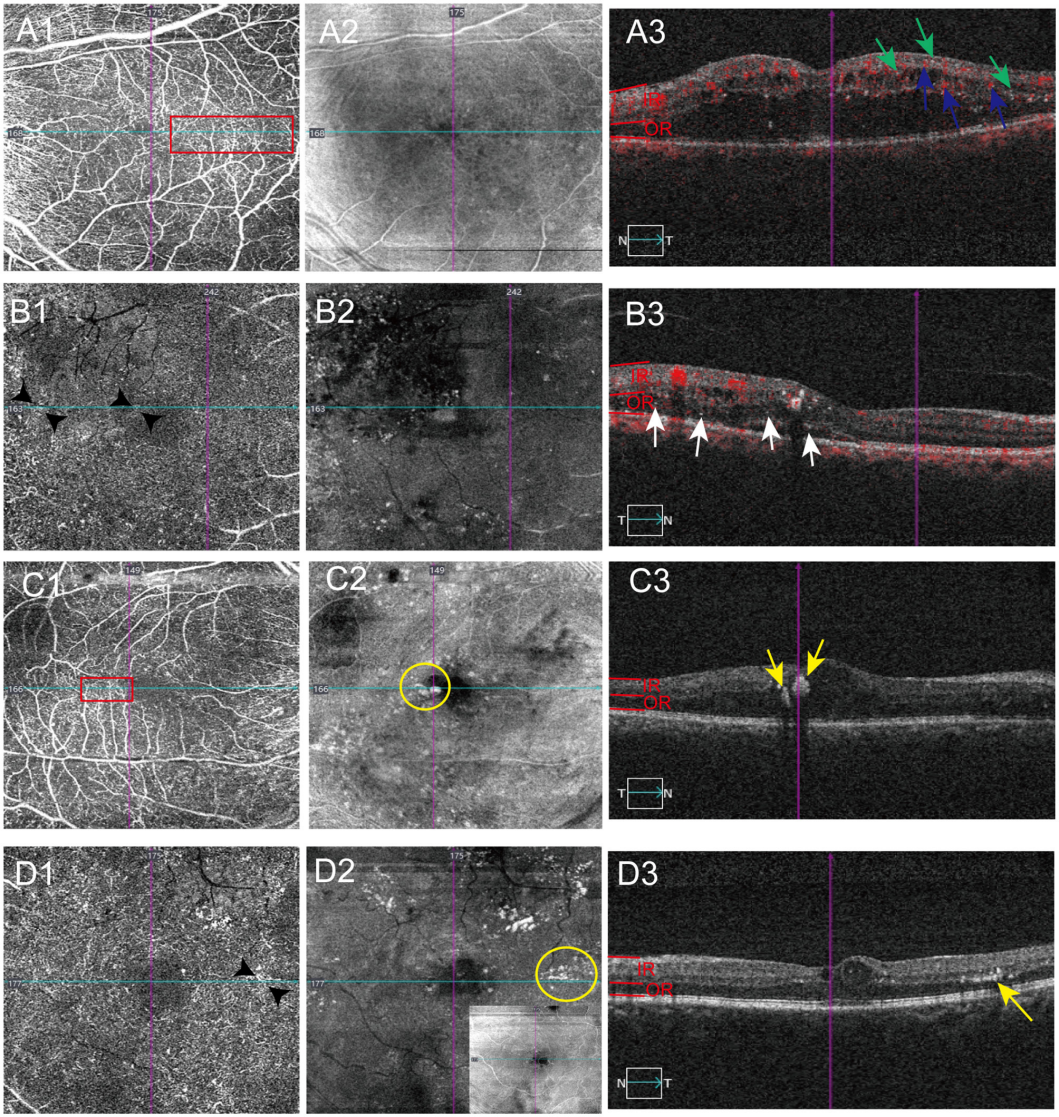
Distribution of hyperreflective materials in the area of intra-retinal microvascular abnormalities (IRMA) detected by OCTA. The scanning position of each image is identified by horizontal (lake blue) or vertical navigation (purple) line. **(A1–A3)** OCTA images from a DME patients show the association of inner retinal hyperreflective spots (IRHFs) and decorrelation-positive hyperreflective materials distribution with the IRMA. Red rectangle showing IRMA in superficial vascular plexuses en face OCTA images **(A1)**, IRHFs (**A3**, green arrows) and decorrelation-positive hyperreflective materials (**A3**, blue arrows) are visible in the corresponding area of OCTA B-scan and invisible in the superficial OCT en face image **(A2)**. **(B1–B3)** Example of outer retinal hyperreflective spots (ORHFs) distributing in the area of IRMA. IRMA is showed in deep vascular plexuses en face OCTA images (**B1**, black arrow heads), ORHFs (**B3**, white arrows) are detected on B scan images and invisible in the deep OCT en face image **(B2)**. **(C1–C3)** OCTA images of a DR patient with inner retinal hard exudates (IRHE) demonstrate IRHE are mostly located on IRMA. IRHE are visible on superficial OCT en face (**C2**, yellow circle) and B scan images (**C3**, yellow arrows), where IRMA are apparently detected by superficial vascular plexuses en face OCTA images (**C1**, red rectangle). **(D1–D3)** OCTA images of a DR patient with outer retinal hard exudates (ORHE). ORHE are observed on deep structural en face (invisible on superficial, **D2**, yellow circle) and B scan images (**D3**, yellow arrows), where IRMA also are detected by deep vascular plexuses en face OCTA images (**D1**, black arrow heads).

Quantitative analysis revealed distinct topographic distributions of hyperreflective materials (HRMs) across four retinal vascular abnormalities visualized by OCTA, with excellent intergrader reliability (Supplementary Table S2). The majority of IRHFs (Table 2, 75.4%, *P* < 0.0001, ICC = 0.991) and ORHFs (Table 2, 89.5%, *P* < 0.0001, ICC = 0.999) colocalized with intraretinal microvascular abnormalities (IRMA), characterized by segmentally tortuous capillaries connecting arterioles and venules in both superficial (Figures 3A1–A3, red rectangle in Figure 3A1 indicating IRMA, green arrows indicating IRHFs) and deep vascular plexuses (Figures 3B1–B3, areas between black arrow heads in Figure 3B1 indicating IRMA, white arrows indicating ORHFs). Non-perfusion areas contained 19.0% of IRHFs (Table 2, *P* < 0.0001, ICC = 0.987) and 8.7% of ORHFs (Table 2, *P* < 0.0001, ICC = 0.997; Figures 2A, C, white arrows); while neovascular membranes showed minimal HRMs association (3.3% IRHFs and 1.4% decorrelation-positive HRMs; Figures 2D–F, red arrow heads in 3D indicating neovascular area, green arrows indicating IRHFs and blue arrows indicating decorrelation-positive HRMs). En face imaging demonstrated that 90.8% of superficial inner retinal hard exudates (Figure 3C2, yellow circle; Figure 3C3, yellow arrows) and 94.0% of deep outer retinal hard exudates (Figure 3D2, yellow circle; Figure 3D3, yellow arrows) precisely corresponded to IRMA locations (Figures 3C1, 3D1, red rectangle and black arrow heads showed IRMA area; Table 2, both *P* < 0.0001, ICC = 0.999), compared to 7.2% and 4.0%, respectively in non-perfusion areas (Figure 2B, yellow arrows). Decorrelation-positive HRMs showed strong spatial correlation with IRMA (Table 2, 77.6%, *P* < 0.0001, ICC 0.930; Figure 3A1 and blue arrows in Figure 3A3) and microaneurysms (Table 2, 21.0%, *P* = 0.003, ICC = 0.954; Figures 2G, H and blue arrows in Figure 2I), while cotton-wool spots exhibited exclusive localization to non-perfusion regions (Figures 2B, C, red arrows and circle).

**Table 2:** Distribution of HRMs in the retinal vascular map of optical coherence tomography angiography.

**HRMs**	**Retinal vascular map of OCTA**				
	**NP**	**IRMA**	**Microneurysm**	**Neovascular**	***P*** **-value**
IRHFs	1,345 (19.0)	5,322 (75.4)	163 (2.3)	232(3.3)	<0.0001
ORHFs	758 (8.7)	7,776 (89.5)	115 (1.3)	39 (0.5)	<0.0001
IRHE	197 (7.2)	2,474 (90.8)	42 (1.5)	11 (0.5)	<0.0001
ORHE	197 (4.0)	4,544 (94.0)	94 (1.9)	5 (0.1)	<0.0001
Decorrelation-positive HRMs	0 (0.0)	6,091 (77.6)	1,648 (21.0)	114 (1.4)	<0.0001
Cotton-wool spot	91 (100.0)	0 (0.0)	0 (0.0)	0 (0.0)	<0.0001

HRMs, hyperreflective materials; IRHFs, inner retinal hyperreflective spots; ORHFs, outer retinal hyperreflective spots; IRHE, inner retinal hard exudates; ORHE, outer retinal hard exudates; NP, no-perfusion; IRMA, intra-retinal microvascular abnormalities.

### Quantitative analysis of hyperreflective materials in DR with and without DME

To investigate the potential role of HRMs in diabetic macular edema (DME), we stratified patients with DR into two groups: those with DME and those without DME. Quantitative analysis revealed significantly higher HRMs counts in DME eyes across all vascular abnormalities (all *P* < 0.05, Table 3). Specifically, inner retinal hyperreflective spots were markedly increased in DME eyes within non-perfusion areas [median 8 (0–72) vs. 0 (0–18), *P* < 0.001], IRMA regions [median 40 (0–140) vs. 0 (0–42), *P* < 0.001], and neovascular areas [median 2 (0–39) vs. 0 (0–9), *P* = 0.002]. Similarly, outer retinal hyperreflective spots showed significant elevation in non-perfusion zones [median 2 (0–39) vs. 0 (0–12), *P* = 0.002], IRMA regions [median 54 (0–216) vs. 0 (0–34), *P* < 0.001], and microaneurysms [median 1 (0–78) vs. 0 (0–6), *P* < 0.001]. Hard exudates demonstrated comparable increases, with inner retinal hard exudates (IRHE) and outer retinal hard exudates (ORHE) being significantly more prevalent in both non-perfusion [IRHE: median 2 (0–20) vs. 0 (0–8), *P* = 0.009; ORHE: median 2 (0–25) vs. 0 (0–5), *P* = 0.013] and IRMA regions [IRHE: median 12 (0–108) vs. 0 (0–50), *P* < 0.001; ORHE: median 24 (0–215) vs. 0 (0–54), *P* < 0.001]. Decorrelation-positive HRMs and cotton-wool spots in non-perfusion areas were also significantly more frequent in DME eyes. Spatial distribution analysis revealed that 46.3% of IRHFs, 51.8% of ORHFs, 56.1% of IRHE, 57.3% of ORHE and 34.8% decorrelation-positive HRMs localized to cystoid space walls, while 11.1%−16.0% of these HRMs subtypes were observed at the outer border of serous retinal detachments (Supplementary Table S1), suggesting preferential accumulation at fluid-tissue interfaces. Among these HRMs, only IRHFs showed a moderate correlation with worse LogMAR acuity (β = 0.1, 95% CI: 0.0–0.2, *P* < 0.001, Supplementary Table S3), indicating a potential functional on visual function.

**Table 3:** Comparison of hyperreflective materials number between DR with DME and DR without DME.

**HRMs**	**Vascular abnormality**	**DR with DME, median (range)**	**DR without DME, median (range)**	***P*-value**
IRHFs	NP	8 (0–72)	0 (0–18)	<**0.001**
	IRMA	40 (0–140)	0 (0–42)	<**0.001**
	Microneurysm	0 (0–23)	0 (0–15)	0.400
	Neovascular	2 (0–39)	0 (0–9)	**0.002**
ORHFs	NP	2 (0–39)	0 (0–12)	**0.002**
	IRMA	54 (0–216)	0 (0–34)	<**0.001**
	Microneurysm	1 (0–78)	0 (0–6)	<**0.001**
	Neovascular	0 (0–11)	0 (0)	0.552
IRHE	NP	2 (0–20)	0 (0–8)	**0.009**
	IRMA	12 (0–108)	0 (0–50)	<**0.001**
	Microneurysm	0 (0–5)	0 (0–7)	0.151
ORHE	NP	2 (0–25)	0 (0–5)	**0.013**
	IRMA	24 (0–215)	0 (0–54)	<**0.001**
	Microneurysm	0 (0–35)	0 (0–9)	0.231
Decorrelation-positive HRMs	IRMA	46 (0–200)	0 (0–37)	<**0.001**
	Microneurysm	0 (0–51)	15 (0–50)	**0.003**
	Neovascular	1 (0–44)	0 (0–4)	<**0.001**
Cotton-wool spot	NP	1 (0–4)	0 (0)	<**0.001**

HRMs, hyperreflective materials; IRHFs, inner retinal hyperreflective spots; ORHFs, outer retinal hyperreflective spots; IRHE, inner retinal hard exudates; ORHE, outer retinal hard exudates; NP, no-perfusion; IRMA, intra-retinal microvascular abnormalities, DR, diabetic retinopathy; DME, diabetic macular edema. Bold values indicate statistically and clinically significant data (*P* < 0.05).

### Correlation of systemic metabolic traits with hyperreflective materials

Our generalized estimating equation (GEE) analysis presented in Table 4 identified significant associations between systemic metabolic parameters and the counts of hyperreflective materials in patients with DR. The duration of diabetes exhibited an inverse correlation with inner retinal hard exudates (IRHE, β = −0.1, 95% CI: −0.2–0.0, *P* = 0.037). Furthermore, the severity of DR demonstrated positive correlations with both inner retinal hyperreflective spots (IRHFs; β = 0.7, 95% CI: 0.4–1.0, *P* < 0.001), outer retinal hyperreflective spots (ORHFs; β = 0.7, 95% CI: 0.2–1.1, *P* = 0.002) as well as cotton-wool spots (β = −0.6, 95% CI: −0.2–1.0, *P* = 0.002). Glycemic control markers exhibited divergent relationships: HbA1c correlated negatively with IRHFs (β = −0.1, 95% CI: −0.2–0.1, *P* < 0.001) and ORHFs (β = −0.1, 95% CI: −0.2–0.1, *P* = 0.01), whereas fasting blood glucose (FBG) showed positive correlations with IRHFs (β = 0.1, 95% CI: 0.0–0.2, *P* < 0.001), ORHFs (β = 0.1, 95% CI: 0.0–0.2, *P* = 0.007), and cotton-wool spots (β = −0.1, 95% CI: 0.0–0.2, *P* = 0.008). Lipid metabolism parameters demonstrated particularly strong associations, with LDL cholesterol showing positive correlations for ORHFs (β = 1.0, 95% CI: 0.1–1.9, *P* = 0.028), IRHE (β = 1.4, 95% CI: 0.5–2.4, *P* = 0.004), ORHE (β = 1.2, 95% CI: 0.2–2.3, *P* = 0.02), and cotton-wool spots (β = 1.4, 95% CI: 0.5–2.4, *P* = 0.004). Conversely, apolipoproteins showed inverse relationships: APO a with IRHFs (β = −2.5, 95% CI: −4.3–0.6, *P* = 0.009) and APO b with ORHFs (β = −3.3, 95% CI: −6.1–0.5, *P* = 0.022), IRHE (β = −4.6, 95% CI: −7.8–1.4, *P* = 0.004), and cotton-wool spots (β = −4.6, 95% CI: −7.8–1.5, *P* = 0.01). Declining renal function, as measured by urinary albumin-creatinine ratio (UCR), correlated with increased IRHFs (β = 0.1, 95% CI: 0.0–0.2, *P* = 0.015) and ORHFs (β = 0.1, 95% CI: 0.0–0.2, *P* = 0.023). No significant associations were observed between HRMs counts and age, leukocyte count, or hypertension status. In general, these findings indicate the crucial role of lipid metabolism in the accumulation of hyperreflective materials.

**Table 4:** Correlation of systemic metabolic traits with HRMs.

**Metabolic traits**	**IRHFs**			**ORHFs**			**IRHE**			**ORHE**			**Decorrelation-positive HRMs**			**Cotton-wool spots**		
	β	**95% CI**	***P*** **-value**	β	**95% CI**	***P*** **-value**	β	**95% CI**	***P*** **-value**	β	**95% CI**	***P*** **-value**	β	**95% CI**	***P*** **-value**	β	**95% CI**	***P*** **-value**
DM duration	0.0	−0.1–0.2	0.057	−0.0	−0.1–0.1	0.09	−0.1	−0.2–0.0	**0.037**	−0.1	−0.2–0.0	0.113	0.0	0.0–0.1	0.452	0.0	−0.1–0.1	0.862
DR severity	0.7	0.4–1.0	<**0.001**	0.7	0.2–1.1	**0.002**	0.6	0.1–1.2	0.072	0.5	−0.1–1.1	0.107	0.2	0.0–0.4	0.076	0.6	−0.2–1.0	**0.002**
HbA1c	−0.1	−0.2–0.1	<**0.001**	−0.1	−0.2–0.1	**0.01**	−0.1	−0.2–0.0	0.107	−0.1	−0.3–0.0	0.153	−0.1	−0.2–0.0	0.065	0.1	−0.1–0.2	0.376
FBG	0.1	0.0–0.2	<**0.001**	0.1	0.0–0.2	**0.007**	0.2	0.1–0.4	0.535	0.0	−0.1–0.1	0.467	0.0	0.0–0.1	0.306	0.1	0.0–0.2	**0.008**
TC	0.1	−0.5–0.7	0.754	−0.2	−0.3–0.2	0.481	−0.2	−0.8–0.5	0.718	−0.4	−1.0–0.2	0.216	−0.1	−0.4–0.2	0.562	0.4	−1.0–1.7	0.594
TG	−0.1	−0.2–0.2	0.843	0.0	−0.3–0.2	0.807	0.1	−0.2–0.4	0.473	0.1	−0.4–0.3	0.873	0.0	−0.1–0.2	0.936	−0.0	−0.4–0.3	0.949
HDL	1.3	−0.2–2.8	0.087	0.3	−1.6–2.2	0.758	0.1	−2.0–2.3	0.893	−0.6	−3.0–1.8	0.631	0.0	−1.1–1.1	0.993	1.2	−1.7–4.3	0.408
LDL	0.4	−0.4–1.2	0.394	1.0	0.1–1.9	**0.028**	1.4	0.5–2.4	**0.004**	1.2	0.2–2.3	**0.02**	0.3	−0.3–0.9	0.331	0.5	−1.0–2.0	0.479
APO a	−2.5	−4.3–0.6	**0.009**	−1.7	−4.1–0.7	0.154	−0.8	−4.0–2.4	0.630	−0.1	−3.4–3.2	0.969	−0.3	−1.6–1.0	0.637	−1.2	−4.8–2.4	0.517
APO b	−2.3	−4.7–0.1	0.068	−3.3	−6.1–0.5	**0.022**	−4.6	−7.8 to −1.4	**0.004**	−3.0	−6.8–0.9	0.132	−0.8	−2.2–0.7	0.295	−3.7	−8.0 to −0.5	0.085
WBC	−0.1	−0.2–0.0	0.119	−0.1	−0.2–0.0	0.934	−0.1	−0.3–0.2	0.596	−0.1	−0.4–0.1	0.289	0.0	−0.1–0.1	0.504	−0.1	−0.4–0.2	0.429
UCR	0.1	0.0–0.2	**0.015**	0.1	0.0–0.2	**0.023**	−0.1	−0.2–0.0	0.097	0.0	−0.1–0.1	0.652	0.0	−0.1–0.1	0.242	−0.0	−0.2–02	0.995

HRMs, hyperreflective materials; IRHFs, inner retinal hyperreflective spots; ORHFs, outer retinal hyperreflective spots; IRHE, inner retinal hard exudates; ORHE, outer retinal hard exudates; DM, diabetes mellitus; DR, diabetic retinopathy; FBG, fasting blood glucose; TC, total cholesterol; TG, triglyceride; HDL, high-density lipoprotein; LDL, low-density lipoprotein; apolipoprotein a and b, APO a and APO b; WBC, white blood cell; UCR, urinary albumin-creatinine ration; CI, confidence interval. Bold values indicate statistically and clinically significant data (*P* < 0.05).

## Discussion

This study systematically investigated the characteristics and spatial distribution of hyperreflective materials (HRMs) in diabetic retinopathy (DR) using OCT angiography (OCTA), while exploring their associations with vascular abnormalities and systemic metabolic dysregulation. By integrating morphological criteria (location, reflectivity, size, back-shadowing, and decorrelation signals) with OCTA features, we identified six distinct HRMs subtypes: inner/outer retinal hyperreflective foci (IRHFs/ORHFs), inner/outer retinal hard exudates (IRHE/ORHE), decorrelation-positive HRMs, and cotton-wool spots. Notably, 75%−94% of HRMs localized to regions of IRMA or microaneurysms, with pronounced enrichment in diabetic macular edema cases. Our findings further linked HRMs prevalence to systemic lipid dysregulation, suggesting their dual origin in blood-retinal barrier (BRB) disruption and metabolic dysfunction.

The conceptualization of intraretinal HRMs—termed hyperreflective dots (HRDs) or foci in prior studies—has undergone substantial refinement since their initial characterization by Bolz et al. (13). Originally described as punctate OCT signals with elevated reflectivity distributed across retinal layers in DME, HRMs have since been implicated in divergent pathophysiological contexts. spectral-domain OCT correlates HRDs with cholesterol crystal deposits, aligning them histologically with hard exudates of DR (19). More recently, OCT angiography (OCTA) imaging from DME patients has detected some degree of non-vascular decorrelation signals called suspended scattering particles in motion-likely representing suspend lipid-laden macrophages within hypoxic microenvironments (14, 20–22). Notably, HRMs phenotypes exhibit disease-specific spatial patterning: in age-related macular degeneration (AMD), these lesions manifest as scattered outer retinal foci adjacent to fluid reservoirs (23), with longitudinal studies documenting their migration toward inner retinal layers during disease progression (24). Such dynamic behavior has prompted hypotheses linking HRMs to trans-differentiation of retinal cells in AMD (25), while histopathological correlations identify them as intraretinal retinal pigment epithelial (RPE) cell (26). This phenotypic plasticity underscores a critical knowledge gap: the absence of universally accepted diagnostic criteria for HRMs across retinal pathologies.

Prior classification frameworks categorized hyperreflective materials (HRMs) into three subtypes based on OCT-derived morphometrics: microglial-like foci (<30 μm, moderate reflectivity), hard exudates (>30 μm, RPE-comparable reflectivity with shadowing), and microaneurysm-associated deposits (>30 μm, inner retinal localization) (9). While this schema improved diagnostic standardization, it overlooked critical vascular-pathological correlations—a limitation addressed in our study through OCT angiography (OCTA)-guided analysis. By synthesizing OCT structural data with OCTA microvascular mapping, we identified six HRMs subtypes, two of which represent novel entities. Firstly, decorrelation-positive HRMs, despite their static appearance on OCT, these lesions exhibited dynamic OCTA signals colocalizing with intraretinal microvascular abnormalities (IRMA) and microaneurysms, distinct from the “suspended scattering particles” associated with hard exudates or lipid-laden macrophages (20, 22, 27). Non-perfusion-restricted cotton-wool spots are regarded as a novel entity, as they share reflectivity profiles with hard exudates while being confined to ischemic zones. Notably, 75%−94% of HRMs spatially correlated with IRMA regions—a finding undetectable through conventional OCT. Furthermore, our dual retinal layer stratification (inner vs. outer) resolved imaging ambiguities: outer retinal HRMs, often occult on fundoscopy, demonstrated precise spatial relationships with choroidal flow alterations on OCTA en face reconstructions. This multimodal approach establishes a more systematic way to analyze the hyperreflective signal and enriched their interpretation.

The ontogeny and pathobiological significance of HRMs remain contentious, with three predominant mechanistic hypotheses emerging from prior research. One of the main hypotheses is that HRMs are considered as aggregation of activated microglia, therefore always are proposed as a biomarker of neuro-retinal inflammation (11, 28, 29). Another hypothesis is that HRMs represent precursors or components of hard exudates, reflecting blood-retinal barrier (BRB) compromise and systemic dyslipidemia (30, 31). This paradigm gains support from established links between hard exudates, BRB disruption, and lipoprotein dysregulation in DME (32, 33). The third hypothesis for the origination is that HRMs are migration of RPE cells or damaged photoreceptor, therefore serving as an indicator of suboptimal therapeutic responses (34, 35). While these models provide fragmented insights, none fully account for HRMs heterogeneity across disease stages. Therefore, any above hypothesis only represents one state of HRMs at defined stage. Our multimodal analysis bridges this gap by demonstrating that 83% of HRMs colocalize with IRMA and exhibit strong correlations with systemic lipid dysmetabolism. This spatial-metabolic coupling suggests a unified pathogenesis: HRMs likely originate from lipoprotein extravasation through incompetent IRMA-associated vessels, compounded by BRB breakdown-induced inflammatory cascades. Although cross-sectional design precludes definitive causal inference, the preferential sequestration of HRMs at sites of active vascular remodeling favors the lipid extravasation hypothesis over purely inflammatory or migratory mechanisms. Longitudinal studies tracking HRMs dynamics against metabolic parameters will be critical to validate this model.

The ultimate aim of studying the HRMs is to direct the individualized treatment of DR. So far, anti-VEGF agents, intravitreal steroid injections or implants, laser and vitrectomy are all considered as the popular therapy (36, 37), however, their treatment outcomes are always not satisfactory. For instance, only 30%−40% DME patients gain improvement of visual acuity 1 year after at least three consecutive monthly anti-VEGF treatment (38, 39). This underscores the need for reliable biomarkers to predict treatment response and guide individualized therapy. HRMs have emerged as a promising candidate biomarker for DME management. A systematic review evaluated the predictive efficacy of HRMs in assessing therapeutic responses, revealing a consistent reduction in HRMs quantity following anti-VEGF therapy or intravitreal steroid implants; the definitive prognostic value of HRMs remains inconclusive, as current evidence does not uniformly establish their role as reliable predictors of treatment success (40). Intriguingly, recent studies suggest that HRM regression after anti-VEGF therapy may correlate with better functional outcomes, supporting their potential role as dynamic indicators of treatment response (41). Notably, in cases exhibiting suboptimal responses to anti-VEGF agents, switching to intravitreal steroids has demonstrated improved clinical outcomes, likely attributable to the potent anti-inflammatory effects of corticosteroids, which more effectively mitigate the inflammatory and vascular permeability pathways implicated in HRMs pathogenesis (42, 43). Beyond their structural significance, HRMs are intrinsically linked to inflammatory and metabolic dysregulation in DME, factors that may critically influence treatment responsiveness. While intravitreal steroids appear particularly beneficial in eyes with higher HRMs burden, clinicians should remain vigilant for the associated risk of more frequent and earlier DME recurrence in these cases. Cumulatively, these observations suggest that HRMs status may serve as a key determinant in therapeutic decision-making, with a stronger predictive association favoring corticosteroid efficacy over anti-VEGF agents. Our findings further expand the therapeutic landscape by proposing lipid-lowering interventions as a complementary strategy, given the well-documented interplay between HRMs and dyslipidemia. Retinal cholesterol accumulation due to impaired lipid metabolism has long been implicated in diabetic retinopathy progression; and recent evidences support the use of lipid-modulating agents—such as fenofibrate, statins, and omega-3 PUFAs—alongside glycemic and blood pressure control to mitigate disease severity (33, 44, 45). In this context, our OCTA-based HRMs classification and its correlation with systemic lipid profiles carry significant clinical relevance, offering a potential avenue for personalized treatment approaches in DME management.

This work has several constraints: (1) cross-sectional design precludes assessment of HRMs evolution or causality; (2) single-timepoint metabolic measures may not capture dynamic interactions with retinal pathology; (3) unmeasured confounders (genetic/epigenetic factors, metabolic memory effects) could influence observed associations. Future studies integrating serial OCTA with multi-omics profiling will clarify HRMs pathophysiology and therapeutic relevance; and (4) the classification of HRMs relies on manual annotation, and although calibrated by expert consensus, there may be interpretation bias particularly for small lesions with poorly defined margins (e.g., intraretinal hyperreflective foci [IRHFs] ≤ 30 μm). Future investigations incorporating serial OCTA imaging with artificial intelligence (AI) image recognition technology will be essential to elucidate the pathophysiological mechanisms and therapeutic implications of HRMs.

## Conclusion

This study establishes a novel OCTA-guided classification system for HRMs in DR, delineating their spatial association with vascular abnormalities (particularly IRMA and microaneurysms) and systemic lipid dysregulation. The dual pathogenesis of HRMs—rooted in blood-retinal barrier disruption and metabolic dysfunction—highlights their potential as biomarkers for disease staging and therapeutic targeting. Our findings advocate for integrating lipid-lowering strategies with conventional DME therapies, proposing a paradigm shift toward personalized interventions that address both vascular and metabolic drivers of DR progression.

## Data Availability

The original contributions presented in the study are included in the article/Supplementary material, further inquiries can be directed to the corresponding author.
