# Supplementary material for: Topographic associations of hyperreflective materials in diabetic retinopathy: a multimodal correlation with microvascular pathology, structural remodeling and systemic metabolic dysregulation
Source: Front Med (Lausanne). 2025 Jul 16;12:1619819. doi: 10.3389/fmed.2025.1619819 (PMC12307468; doi:10.3389/fmed.2025.1619819)
Supplement: Supplementary file 1 [file Table_1.docx]

**Supplement Table 1** Distribution of hyperreflective materials in the space of diabetic macular edema

|  | Total in DME | Intra-cystoid | Wall of cystoid | Next to SRD |
| --- | --- | --- | --- | --- |
| IRHFs | 6405 | 111 (1.7%) | 2964 (46.3%) | 920 (14.4%) |
| ORHFs | 8232 | 69 (0.8%) | 4264 (51.8%) | 1315 (16.0%) |
| IRHE | 2445 | 70 (2.9%) | 1371 (56.1%) | 374 (15.3%) |
| ORHE | 4562 | 104 (2.3%) | 2614 (57.3%) | 696 (15.3%) |
| Decorrelation-positive HRMs | 7921 | 183 (2.3%) | 2757 (34.8%) | 876 (11.1%) |
| Cotton spot | 88 | 0 | 0 | 0 |

HRMs, hyperreflective materials; IRHFs, inner retinal hyperreflective spots; ORHFs, outer retinal

hyperreflective spots; IRHE, inner retinal hard exudates; ORHE, outer retinal hard exudates;

DME, diabetic macular edema; SRD, serous retinal detachment.

| HRMs | Intraclass Correlation Coefficient between intergrader, ICC | | | |
| --- | --- | --- | --- | --- |
|  | NP | IRMA | Microneurysm | Neovascular |
| IRHFs | 0.987 | 0.991 | 0.994 | 0.999 |
| ORHFs | 0.997 | 0.999 | 0.999 | 0.997 |
| IRHE | 0.998 | 0.999 | 0.987 | 0.957 |
| ORHE | 0.993 | 0.998 | 0.998 | 0.999 |
| Decorrelation-positive HRMs | - | 0.930 | 0.954 | 0.977 |
| Cotton-wool spot | 0.990 | - | - | - |

**Supplement Table 2** Intraclass Correlation Coefficient for intergrader agreement

HRMs, hyperreflective materials; IRHFs, inner retinal hyperreflective spots; ORHFs, outer retinal

hyperreflective spots; IRHE, inner retinal hard exudates; ORHE, outer retinal hard exudates; NP, no-perfusion;

IRMA, intra-retinal microvascular abnormalities.

**Supplement Table 3** Correlation of HRMs with visual acuity

|  | Visual acuity (LogMAR) | | |
| --- | --- | --- | --- |
|  | *β* | 95% CI | *P* |
| IRHFs | 0.1 | 0.0-0.2 | **＜0.001** |
| ORHFs | 0.0 | -0.01-0.00 | 0.74 |
| IRHE | 0.0 | -0.01-0.00 | 0.513 |
| ORHE | 0.0 | -0.01-0.00 | 0.186 |
| Decorrelation-positive HRMs | 0.0 | -0.01-0.00 | 0.079 |
| Cotton-wool spots | 0.0 | -0.1-0.1 | 0.828 |

HRMs, hyperreflective materials; IRHFs, inner retinal hyperreflective spots; ORHFs, outer retinal hyperreflective spots;

IRHE, inner retinal hard exudates; ORHE, outer retinal hard exudates; CI, confidence interval.
